# Less frequent radiological exams to avoid futile response assessments from 177-LuDOTATE therapy for patients with advanced neuroendocrine tumors

**DOI:** 10.1530/EO-24-0021

**Published:** 2024-10-29

**Authors:** Carolina C Marques, Angelo B Brito, Eduardo N Lima, Mauro D Donadio, Rachel P Riechelmann

**Affiliations:** 1 AC Camargo Cancer Center São Paulo, Brazil; 2 Centro Paulista de Oncologia – Oncoclinicas, São Paulo, Brazil

**Keywords:** 177-LuDOTATE, neuroendocrine tumors, radiological response

## Abstract

**Background:**

177-LuDOTATE is an effective but expensive treatment for neuroendocrine tumors (NETs). Reducing treatment-related costs, such as the number of images, could improve access to 177-LuDOTATE. We evaluated early radiological tumor progression and prognostic factors in patients with NETs treated with 177-LuDOTATE.

**Methods:**

We retrospectively included all patients with NETs who received at least one cycle of 177-LuDOTATE. The primary endpoint was the rate of early radiological progression between cycles 2 and 3 (in 10–16 weeks). Secondary endpoints were progression-free survival (PFS) and overall survival (OS) according to prognostic factors (tumor grade, primary site, functioning syndrome, 177-LuDOTATE treatment line) in Cox proportional hazards models.

**Results:**

The median number of 177-LuDOTATE cycles was 3 (range 1–6) among 59 patients included. Ten (17%) patients had early progression. Among 14 patients who received ≤2 cycles of 177-LuDOTATE, ten (72%) stopped treatment due to disease progression, with five patients having a G2 (ki67: 5–25%) and 4, a G3 (ki67: 25–90%) NET. In the Cox multivariable analysis, higher grade (G2 or G3 vs G1) were significantly associated with inferior PFS and OS. The median PFS of G1, G2 and G3 NET patients were: 34.1, 11.7 and 6.1 months (*P* = 0.015), respectively.

**Conclusions:**

It is feasible to perform imaging tests after 177-LuDOTATE completion for patients with indolent NETs with the intent to avoid futile assessments. For patients with more aggressive diseases, such as G3 NETs and G2 tumors with high tumor burden, we advise to perform more frequent images during 177-LuDOTATE therapy.

## Introduction

The treatment of patients with neuroendocrine tumors (NETs) often requires a multidisciplinary approach, incorporating medical, surgical and interventional strategies aimed at disease control and symptom management. Therapeutic interventions for well-differentiated unresectable or metastatic NETs encompass somatostatin analogs (SSA), liver-directed therapies, targeted therapy, peptide receptor radionuclide therapy (PRRT) with 177-LuDOTATE, and cytotoxic chemotherapy, typically reserved for more aggressive tumors (Riechelmann *et al.* 2023).

The efficacy and safety of PRRT for patients with advanced NETs are substantiated by extensive retrospective and phase III clinical trials. The NETTER-1 phase III trial (Strosberg *et al.* 2017) exhibited a significant improvement in median PFS in patients with progressive G1/G2 midgut advanced NETs treated in second line with 177-LuDOTATE compared with octreotide long acting release (LAR) 60 mg monthly, from 8.5 to 25 months. Recent data from the phase III NETTER-2 trial, which compared the efficacy of 177-LuDOTATE and octreotide LAR 30 mg monthly with octreotide LAR 60 mg monthly in first line for patients with advanced G2 or G3 gastroenteropancreatic NETs, also showed a significant gain in median PFS, from 8.5 months to 22.8 months with 177Lu-DOTATATE (Singh *et al.* 2024). In these phase III trials, 177-LuDOTATE is administered every 8 weeks for up to four doses.

Despite its proven efficacy, 177-LuDOTATE is not available in many countries because of its high cost and logistics of delivering and/or production. A less expensive approach is its local production by many countries, including Brazil, where PRRT is produced by the government-funded Institute of Nuclear Medicine Research. Minimizing costs associated with therapies and hospital visits, and avoiding futile interventions, is important to improve access to cancer care. We hypothesized that fewer imaging tests could be feasible in patients with more indolent NETs undergoing 177-LuDOTATE. Therefore, we evaluated the rate of early radiological progression and prognostic factors for tumor progression in these patients.

## Methods

We performed a retrospective study of all adult patients with histologically confirmed advanced NETs treated at AC Camargo Cancer Center, São Paulo, Brazil. Eligible patients who had metastatic/inoperable NETs of any origin and who received at least one cycle of 177-LuDOTATE, had a radiological evaluation of response and were followed up in our institution. In our institution, it is routine practice to perform radiological evaluation every two cycles of 177-LuDOTATE, e.g. between cycles 2 and 3 and after cycles 4 (every 10–16 weeks), following phase III trials of 177-LuDOTATE (Strosberg *et al.* 2017).

The following clinical and demographic data were extracted from electronic medical records: age at the time of diagnosis, sex, anatomical and pathological variables (tumor grade, Ki67 index, number of metastatic sites), presence of carcinoid syndrome or other hormone-related syndromes, previous treatments, line of treatment with 177-LuDOTATE, number of cycles at 177-LuDOTATE, radiological response evaluations and follow-up status.

Overall survival (OS) was defined as the time from the start of C1 177-LuDOTATE to the date of the last follow-up or death. Progression-free survival (PFS) was defined as the time from C1 of 177-LuDOTATE to date disease progression or death. The median time since diagnosis of metastatic disease was defined as the median time from the date of diagnosis of metastatic disease to C1 of 177-LuDOTATE. Response assessments were carried out by consulting image reports with measurement of tumor lesions as described in medical records. Disease progression was considered as the appearance of a new lesion and/or an increase of at least 20% in the largest diameter of targeted lesions on CT and/or MRI of the chest, abdomen and pelvis.

Collected data were tabulated and statistically analyzed. Absolute values and ratios were used to describe the distributions of categorical variables. Time-to-event variables were estimated by the Kaplan–Meier method and were compared using the long-rank test. The Cox regression proportional hazard multivariable model was used to adjust for prognostic variables of OS and PFS (histological grade, primary site, functioning syndrome and 177-LuDOTATE line of treatment). Multivariate analysis was performed only with variables that presented a *P* value <0.20 in the univariate analysis. For multivariate analysis, we considered *P* values <0.05. Statistical analysis was performed with SPSS software, version 24 (2016).

## Results

From November 2006 to March 2023, 80 patients were selected but 21 were excluded because of insufficient information on Ki67 and/or histological grade. Hence, 59 patients were included for analysis: 19 (32%) had G1 NETs, 33 (56%) had G2 NETs and 7 (12%) had G3 tumors. Their median age was 52 years (27–78 years), and most were male (60%). The most common primary tumor locations were the small bowel (46%) and pancreas (34%). The majority (52.5%) of patients received 177-LuDODATE in the second line; 13 (22%) patients received 177-LuDODATE in the first line; and 15 (25.5%) patients received it in three or more lines of treatment. Fourteen patients had only two or fewer cycles of 177-LuDOTATE, mostly (*n* = 10; 72%) because of disease progression during therapy. Four patients had to prematurely discontinue 177-LuDOTATE because of persistent pancytopenia, national shortage of 177-LuDOTATE, treatment-related renal dysfunction and clinical deterioration (frail old patient with exacerbation of previous comorbidities). Table 1 summarizes the characteristics of patients.

**Table 1:** Baseline characteristics of patients.

Clinical and pathological characteristics	No. of patients (*n* = 59; 100%)
Gender	
Male	35 (60)
Female	24 (40)
Age	
Median, range in years	52 (27–78)
Median time since diagnosis of metastatic disease (years)	2.58
Histological grade	
Grade 1	19 (32)
Grade 2	33 (56)
Grade 3	7 (12)
Primary site	
Pancreas	20 (34)
Small bowel	27 (46)
Others	12 (20)
Primary tumor resection	
Yes	39 (66)
No	20 (34)
Number of treatment modalities before 177Lu-DOTATATE	
≤1	13 (22)
2	31 (52.5)
≥3	15 (25.5)
Metastasis sites	
Liver	49 (83)
Lymph nodes	33 (55)
Bone	6 (10
Peritoneum	10 (17)
Others	13 (22)
Number of 177-LuDOTATE doses	
Two of fewer	14 (24)
Three or more	45 (76)
Functioning syndrome	
Yes	21 (36)
No	38 (64)

In imaging response assessment between cycles 2 and 3, 30 patients (57.7%) showed a partial response; 12 patients (23.1%) had stable disease and 10 patients (19.2%) experienced progressive disease (PD).

Of those patients who had early PD, 40% had a functioning syndrome, 50% had small bowel, 20% lung, 20% pancreatic and 10% colorectal origin. Regarding histological grade, 10%, 50% and 40% had grades 1, 2 and 3, respectively. Among the four G3 cases, ki67 index was 25% in three tumors and 60% in one. Among five patients with G2 tumors, three had NETs with ki67 index of 4%, 5% and 5%, and two patients had tumors ki67 of 10%. These patients had aggressive disease, with high tumor burden (bulky hepatic metastases). Four had aggressive hormonal syndromes by the time of PRRT initiation: three with refractory carcinoid syndrome and one with an adrenocorticotrophin hormone-producing pancreatic NET.

**Figure 1:**
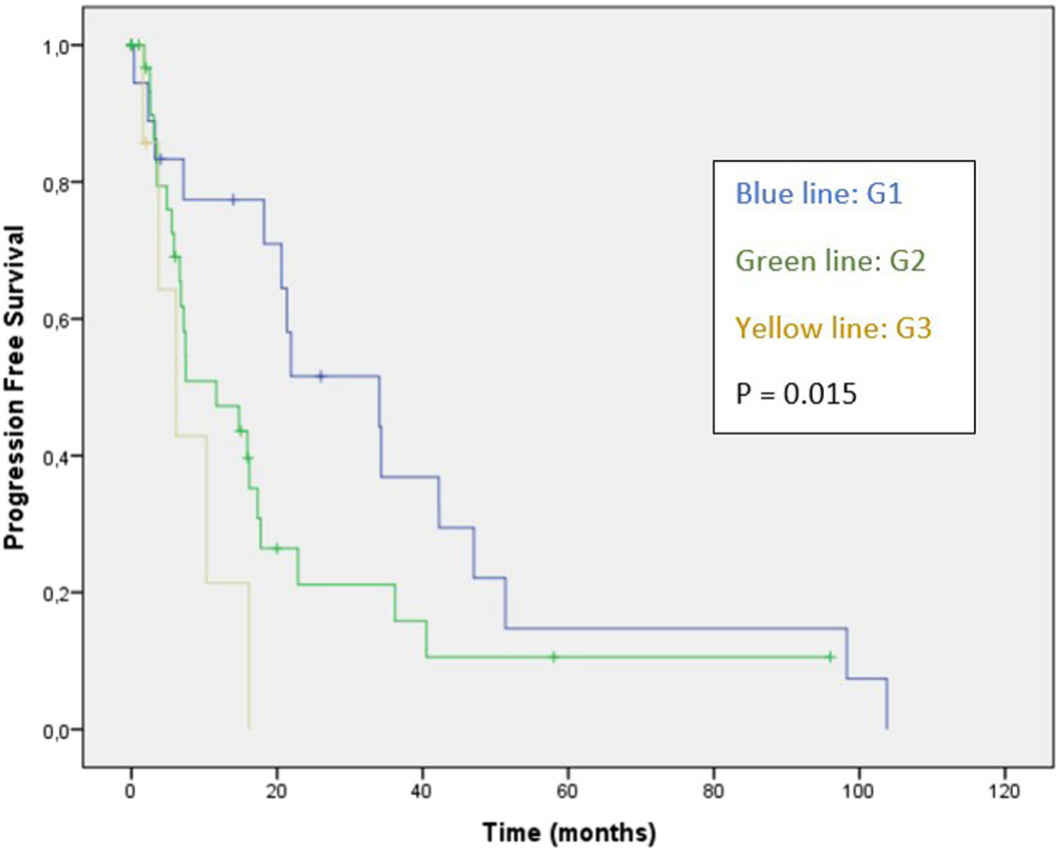
Progression-free survival according to histological grade.

The median OS for all patients was 43.7 months (95% CI: 29.5–57.8), and the median PFS was 16.1 months (95% CI: 8.76–23.59). Cox analysis for OS shows non-significant worse outcomes for non-G1 tumors, presence of carcinoid syndrome and later lines of 177-LuDOTATE (Table 2). G3 NETs were associated with inferior PFS in univariate (hazard ratio (HR): 4.46; 95% CI: 1.47–13.94; *P* = 0.008) and multivariate analysis (HR: 4.46; 95% CI: 1.47–13.94; *P* = 0.008), respectively (Table 3). Median PFS for G1, G2 and G3 NET patients were 34.1, 11.7 and 6.1 months, respectively (*P* = 0.015; Fig. 1).

**Table 2:** Unadjusted and adjusted analyses of overall survival.

Cox analysis	Univariate analysis		Multivariate analysis	
Histological grade	HR (95% CI )	*P*	HR (95% CI)	*P*
G1	1.00		1.00	
G2	2.06 (0.95–4.47)	0.06	2.14 (0.95–4.64)	0.06
G3	2.93 (0.89–9.70)	0.07	2.23 (0.61–8.14)	0.22
Primary site				
Small bowel	1.00			
Pancreas	1.44 (0.68–3.01)	0.33	NA	
Others	1.54 (0.64–3.71)	0.33	NA	
Functioning syndrome				
No	1.00			
Yes	1.75 (0.83–3.70)	0.14	1.93 (0.91–4.09)	0.15
177-LuDOTATE line				
≤2	1.00			
>2	1.87 (0.88–3.98)	0.10	1.79 (0.78–4.04)	0.16

G, grade; HR, hazard ratio.

**Table 3:** Unadjusted and adjusted of progression-free survival.

Cox analysis	Univariate analysis		Multivariate analysis	
Histological grade	HR (95% CI)	*P*	HR (95% CI )	*P*
G1	1.0		1.00	
G2	1.90 (0.94–3.82)	0.07	1.90 (0.94–3.82)	0.07
G3	4.46 (1.47–13.94)	0.008	4.46 (1.47–13.94)	0.008
Primary tumor				
Small bowel	1.0			
Pancreas	0.94 (0.41–2.17)	0.90	NA	
Others	0.85 (0.36–2.05)	0.73	NA	
Functioning syndrome				
No	1.00		NA	
Yes	1.07 (0.57–1.99)	0.83	NA	
177-LuDOTATE line				
≤2	1.00		NA	
>2	1.40 (0.66–2.96)	0.37	NA	

G, grade; HR, hazard ratio.

Seven patients had G3 NETs, which were analyzed in detail for imaging response and clinical evolution. Of these, five patients had disease progression confirmed on imaging in the first evaluation between cycles 2 and 3, and two showed a partial response.

## Discussion

In this retrospective unicenter study, we observed that patients with advanced G1 or indolent G2 NETs rarely present early progression (in 10–16 weeks) after initiation of 177-LuDOTATE. In fact, the median PFS was 34.1 for patients with G1 NETs and 11.7 months for those with G2 NETs. Therefore, we think it is feasible to skip radiological imaging tests in 8 to 10 weeks for patients with indolent NETs, potentially saving costs and avoiding unnecessary exams by reserving imaging for post-treatment completion. However, for patients with more aggressive diseases, such as those with G3 NETs or even G2 tumors with aggressive bevahior, such as more rapid growth and/or bulky disease, it is prudent to perform more frequent imaging tests during treatment with 177-LuDOTATE.

The majority of our G3 NETs patients demonstrated progression after cycle 2, resulting in notably inferior PFS. A European retrospective multicenter study, examining PRRT treatment in 114 patients with grade 3 gastroenteropancreatic NETs, showed that 20% presented PD after completing PRRT treatment and that those with tumors with higher proliferation index (Ki-67 ≥55%) displayed PD in 43% of cases (Carlsen *et al.* 2019). Our study revealed that four out of ten patients who experienced early progression had G3 NETs. Yet, in our cohort, 15% of patients with G2 NETs (5 out of 33) experienced early PD. These patients had more aggressive disease, with more pronounced tumor bulky and/or uncontrolled functioning syndrome at the time of PRRT initiation.

Because of their longer PFS, patients with G1 NETs might permit delayed response assessments during therapies such as 177-LuDOTATE, potentially lowering healthcare expenses. Cost-effectiveness analyses have indicated that 177-LuDOTATE for unresectable/metastatic progressive GEP-NETs is associated with improved quality-adjusted life expectancy compared with best supportive care in patients with small bowel NETs (using the NETTER-1 trial data) or with everolimus or sunitinib in pancreatic NETs (Mujica-Mota *et al.* 2018, Leeuwenkamp *et al.* 2020, Palmer *&* Leeuwenkamp 2020, Smith-Palmer *et al.* 2021). However, healthcare resource allocation in NETs is still high.

Data collected from Swedish national registries, encompassing 478 patients with metastatic G1 and G2 GEP-NETs prior to the era of 177-LuDOTATE, assessed healthcare resource utilization (HRU) and the associated cost of illness. Direct costs accounted for 77%, while costs attributed to production loss comprised 22%. Among the direct medical costs, prescription drugs emerged as the primary contributor (Lesén *et al.* 2019). Additionally, an HRU and treatment practice analysis study, incorporating data from the United States, United Kingdom, Germany, France, Brazil, and Italy, revealed that advanced NETs are associated with substantial resource utilization across various subtypes and countries.

Disease progression correlates with heightened utilization of chemotherapy, hospitalization and targeted therapy (Casciano *et al.* 2013). Overall, HRU in advanced NETs in the United States is considerable, with data indicating that disease progression impacts resource utilization irrespective of the site of the NET tumor. Multivariate analysis from a retrospective study involving 110 patients revealed that during the first progression period, patients were more than three times as likely to undergo chemotherapy compared to baseline (odds ratio (OR): 3.31; 95% CI: 1.46–7.48, *P* = 0.0041). Additionally, progression was linked to a higher probability of having a study physician visit (relative risk (RR): 1.54; 95% CI: 1.10–2.17, *P* = 0.0117), as well as an increased frequency of other physician visits (RR: 1.84; 95% CI: 1.10–3.10, *P* = 0.0211) (Strosberg *et al.* 2013). Therefore, effective treatments theoretically reduce costs when they delay progression. Yet, patients still need to perform frequent tests during this disease monitoring, which suggests that strategies to reduce HRU during follow-up are also warranted.

HRU escalates further when considering the presence of carcinoid syndrome. The total resource costs may increase by nearly 40% when comparing controlled and uncontrolled carcinoid syndrome. Costs per patient are predominantly influenced by the use of somatostatin analogs, medical interventions and examinations (Lesén *et al.* 2018). In a multicenter retrospective study involving 137 consecutive patients with NETs and elevated urinary 5-HIAA (hydroxy indole acetic acid) treated at seven large hospitals in Latin America, NET patients with carcinoid heart disease, a known complication from carcinoid syndrome, exhibited higher HRU independently of other clinical factors or health systems (Tanaka *et al.* 2020).

Another important aspect is the negative impact of futile interventions on patients’ psychological health. Unnecessary radiological exams during PRRT require hospital visits and medical consultations, with the consequent necessity of hours away from work/home. Last but not least, the expected anxiety associated with the results of such exams. Therefore, reducing futile tests and interventions are crucial to providing good clinical care.

While our study contributes valuable insights, it bears limitations inherent to retrospective analyses. Our sample size is modest, and some data were unavailable for analysis, for example, the digital radiological images and accurate symptom information; additionally, tumor tissues were not available for pathology review. We did not evaluate direct costs from the radiological exams or the estimated savings from skipping them. Nevertheless, our study brings relevant information about a simple strategy to avoid futile radiological tests (and all the unnecessary logistics associated with them) and potentially minimize healthcare costs.

Reducing healthcare expenditures is important to expand access to effective therapies for patients’ NETs, particularly with the adoption of newer and pricier therapies like 177-LuDOTATE. Minimizing unnecessary imaging tests during cancer treatments not only benefits society in terms of reducing costs associated with cancer care but also individual patients, reducing their burden. Not less importantly, the psychological distress of more exams, hospital visits and anxiety associated with expectations of results of radiological tests negatively impact patients’ quality of life.

## Conclusion

Based on the data presented, we consider it feasible to omit imaging tests every 10–16 weeks, between cycles 2 and 3, of 177-LuDOTATE in patients with indolent NETs. We think it is appropriate, for these patients, to have imaging testes performed after 177-LuDOTATE completion. This resource-saving strategy is especially important in developing nations. Nevertheless, patients with more aggressive NETs, such as those with G3 NETs and those with G2 tumors with high tumor burden and/or aggressive hypersecretion syndromes, should have imaging tests performed more frequently as they are more likely to present PD during 177-LuDOTATE.

## Declarations of interest

The study was reviewed and approved by IRB – registration number 66057722.7.0000.5432

RPR has received honoraria and consultancy fees from Ipsen. MD has received honoraria and consultancy fees from Ipsen. The other authors declare that they have no conflicts of interest related to the publication of this manuscript.

## Funding

This work did not receive any specific grant from any funding agency in the public, commercial or not-for-profit sector.

## Warnings

The opinions expressed in the report presented are those of the authors and do not necessarily represent the official position of the institution to which they belong.

## Author contribution statement

All authors have made a significant contribution to this manuscript, have seen and approved the final manuscript and agree to its submission to the Endocrine Oncology.

## References

[bib1] Carlsen EA Fazio N Granberg D Grozinsky-Glasberg S Ahmadzadehfar H Grana CM Zandee WT Cwikla J Walter MA Oturai PS *et al.* Peptide receptor radionuclide therapy in gastroenteropancreatic NEN G3: a multicenter cohort study. Endocrine-Related Cancer 2019 26 227–239. (10.1530/ERC-18-0424) 30540557

[bib2] Casciano R Wangc X Stern L Parikh R Chulikavit M Willet J Liu Z Strosberg J Cadiot G & Riechelmann R. International practice patterns and resource utilization in the treatment of neuroendocrine tumors. Pancreas 2013 42 339–347. (10.1097/MPA.0b013e31826707cc) 23357923

[bib3] Leeuwenkamp O Smith-Palmer J Ortiz R Werner A Valentine W Blachier M & Walter T. Cost-effectiveness of lutetium [177Lu] oxodotreotide versus best supportive care with octreotide in patients with midgut neuroendocrine tumors in France. Journal of Medical Economics 2020 23 1534–1541. (10.1080/13696998.2020.1830286) 32990484

[bib4] Lesén E Björstad Å Björholt I Marlow T Bollano E Feuilly M Marteau F Welin S Elf AK & Johanson V. Real-world treatment patterns, resource use and costs of treating uncontrolled carcinoid syndrome and carcinoid heart disease: a retrospective Swedish study. Scandinavian Journal of Gastroenterology 2018 53 1509–1518. (10.1080/00365521.2018.1531653) 30449217

[bib5] Lesén E Granfeldt D Houchard A Berthon A Dinet J Gabriel S Björstad Å Björholt I Elf AK & Johanson V. Cost-of-illness of metastatic gastroenteropancreatic neuroendocrine tumours in Sweden-A population-based register-linkage study. European Journal of Cancer Care 2019 28 e12983. (10.1111/ecc.12983) 30652364 PMC9285913

[bib6] Mujica-Mota R Varley-Campbell J Tikhonova I Cooper C Griffin E Haasova M Peters J, Lucherini S Talens-Bou J Long L, *et al.* Everolimus, lutetium-177 DOTATATE and sunitinib for advanced, unresectable or metastatic neuroendocrine tumours with disease progression: a systematic review and cost-effectiveness analysis. Health Technology Assessment 2018 22 1–326. (10.3310/hta22490) PMC6151360 30209002

[bib7] Palmer J & Leeuwenkamp OR. Cost-effectiveness of lutetium (177Lu) oxodotreotide vs everolimus in gastroenteropancreatic neuroendocrine tumors in Norway and Sweden. World Journal of Clinical Cases 2020 8 4793–4806. (10.12998/wjcc.v8.i20.4793) 33195647 PMC7642527

[bib8] Riechelmann RP Taboada RG de Jesus VHF Iglesia M & Trikalinos NA. Therapy sequencing in patients with advanced neuroendocrine neoplasms. American Society of Clinical Oncology Educational Book. American Society of Clinical Oncology. Annual Meeting 2023 43 e389278. (10.1200/EDBK_389278) 37257140

[bib14] Singh S Halperin D Myrehaug S Herrmann K Pavel M Kunz PL Chasen B Tafuto S Lastoria S Capdevila J [177Lu]Lu-DOTA-TATE plus long-acting octreotide versus high‑dose long-acting octreotide for the treatment of newly diagnosed, advanced grade 2-3, well-differentiated, gastroenteropancreatic neuroendocrine tumours (NETTER-2): an open-label, randomised, phase 3 study. Lancet 403 2807-2817. (10.1016/S0140-6736(2400701-3) 38851203

[bib10] Smith-Palmer J Leeuwenkamp OR Virk J & Reed N. Lutetium oxodotreotide (177Lu-Dotatate) for the treatment of unresectable or metastatic progressive gastroenteropancreatic neuroendocrine tumors: a cost-effectiveness analysis for Scotland. BMC Cancer 2021 21 10. (10.1186/s12885-020-07710-7) 33402120 PMC7786468

[bib11] Strosberg J Casciano R Stern L Parikh R Chulikavit M Willet J Liu Z Wang X & Grzegorzewski KJ. United States-based practice patterns and resource utilization in advanced neuroendocrine tumor treatment. World Journal of Gastroenterology 2013 19 2348–2354. (10.3748/wjg.v19.i15.2348) 23613628 PMC3631986

[bib12] Strosberg J El-Haddad G Wolin E Hendifar A Yao J Chasen B Mittra E Kunz PL Kulke MH Jacene H, *et al.* NETTER-1 TrialInvestigators. Phase 3 trial of ^177^Lu-Dotatate for midgut neuroendocrine tumors. New England Journal of Medicine 2017 376 125–135. (10.1056/NEJMoa1607427) 28076709 PMC5895095

[bib13] Tanaka H Uema D Rego JFM Weschenfelder RF D'Agustini N Filho DRR O'Connor JM Luca R Nuñez JER de Barros E Silva MJ, *et al.* Health resource utilization by patients with neuroendocrine tumours with or without carcinoid heart disease: a multinational study. Ecancermedicalscience 2020 14 1141. (10.3332/ecancer.2020.1141) 33343700 PMC7738266

